# Effect of Salts on the Aggregation and Strength of Protein-Based Underwater Adhesives

**DOI:** 10.1021/acsomega.5c07638

**Published:** 2025-11-05

**Authors:** Zachary D. Lamberty, Chloe M. Skogg, Michael C. Wilson, Maryssa A. Beasley, Abdon A. Vivas Tejada, Beulah A. Peters, Christopher R. So, Elizabeth A. Yates

**Affiliations:** † 311308 US Naval Research Laboratory, 4555 Overlook Ave SW, Washington, District of Columbia 20375, United States; ‡ United States Naval Academy, 572M Holloway Road, Annapolis, Maryland 21402, United States

## Abstract

While hydrophobic underwater adhesives have often been desired for their ability to remove water from interfaces, their inherent immiscibility with water can also hinder their use. Water-based adhesive systems can lead to improved wetting, lower toxicity, and exhibit dynamic physical responses to aqueous chemistries in the environment. For protein-based adhesives, simple aqueous salts can dramatically alter the intra- and intermolecular forces driving interactions between proteins and with surfaces. Here, we investigate the effect of four main salts found in seawater, NaCl, KCl, MgCl_2_, and CaCl_2_ on underwater curing adhesives made from two agricultural byproduct proteins, bovine serum albumin (BSA), and bovine α-Lactalbumin (αLa). We demonstrate that salts can significantly impact the adhesion of protein-based adhesives, increasing bond strength at moderate salt concentrations but decreasing at higher concentrations. Calorimetry and rheology experiments show that high ionic strengths hasten gelation time to form weaker materials with lower adhesion, while moderate salt concentrations slow protein aggregation to produce stiffer materials with higher bond strengths. The addition of silica fillers increased the bond strength of salt-containing αLa gels but decreased the bond strength of BSA gels. In general, salts that stabilized native protein structures formed stiffer gel networks but tended to decrease adhesion compared to salts with destabilizing effects. When combining simple salts and protein-based adhesives, we demonstrate control over nearly all attributes of adhesive curing and strength as an effective means to improve underwater adhesion.

## Graphical abstract

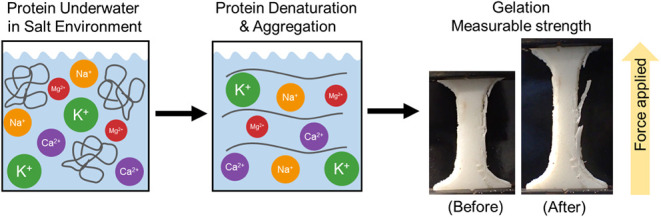

## 1 Introduction

Conventional wisdom dictates that effective underwater adhesion results from the exclusion of water at submerged contacts, leading many researchers to utilize hydrophobic polymers as underwater adhesives.^1–5^ Though counterintuitive, it has recently been demonstrated that aqueous hydrogel adhesives can also adhere underwater at comparable strengths to hydrophobic adhesives.^6^ Hydrogel adhesives, particularly when made from proteins or other biomaterials, can offer improved underwater wetting of surfaces and lower toxicity compared to solvents found in conventional adhesives.^1,7–9^ Additionally, hydrogels facilitate exchange with their solution environment, leading to physical changes in the material and its interaction with surfaces.^10^ Aqueous chemistries such as salts, metal ions, and hydrophilic solutes are all compatible with hydrogels and can be used to control adhesive or bulk interactions.^11,12^

Underwater adhesives are often used in saline environments of varying ionic strength and composition, e.g., in marine or biomedical environments. With hydrogel adhesives, ionic strength affects the swelling of gels and can screen electrostatic interactions that drive adhesion.^1,13–15^ In complex coacervates, drops in ionic strength often drive phase separation and the formation of a cohesive network.^16^ At the same time, hydrated ions in salt water create a boundary layer that is particularly challenging to remove from interfaces before bonding.^17^ Understanding the effect of salt concentration on underwater adhesives is crucial for creating robust and long-lasting bonds.

Though proteins have been used as adhesives for millennia,^18^ there has been recent growing use for these materials in underwater adhesives. Natural adhesive proteins from marine organisms such as mussels, barnacles, or tubeworms are desirable but challenging to scale,^9,19–21^ thus developing methods to process low-cost proteins into biomimetic adhesives is a promising route.^22^ Agricultural byproducts, for example, can be sourced in large quantities and methods have been developed to aggregate them into amyloid fibrils,^23–26^ structures that mimic those found in the adhesive of marine barnacles.^27^ As such, a direct route toward scalable underwater adhesives can be taken, but many questions remain about how aqueous chemistries govern the curing of such an adhesive. Creating an underwater adhesive from byproduct proteins involves converting a normally water-soluble protein into a sticky insoluble material through aggregation. To accomplish this intramolecular bonds of the native protein are disrupted through the use of denaturants^28,29^ and reducing agents^30^ that lead unfolded proteins to aggregate into a complex gel network.

We recently developed an adhesive inspired by barnacles from bovine serum albumin (BSA), which can cure underwater at room temperature through chemical denaturation.^6^ A denaturant and cationic surfactant induce the protein to partially denature into a molten globular state, where a thiol then further reduces disulfide bonds to unfold the protein. In highly concentrated protein solutions, the unfolded proteins will form stable insoluble aggregate structures that combine into a hydrogel network (Figure 1a). This approach is applicable to a wide range of proteins and, as the denaturation and aggregation of proteins occur in water, a broad range of chemicals can be considered.

**Figure 1:**
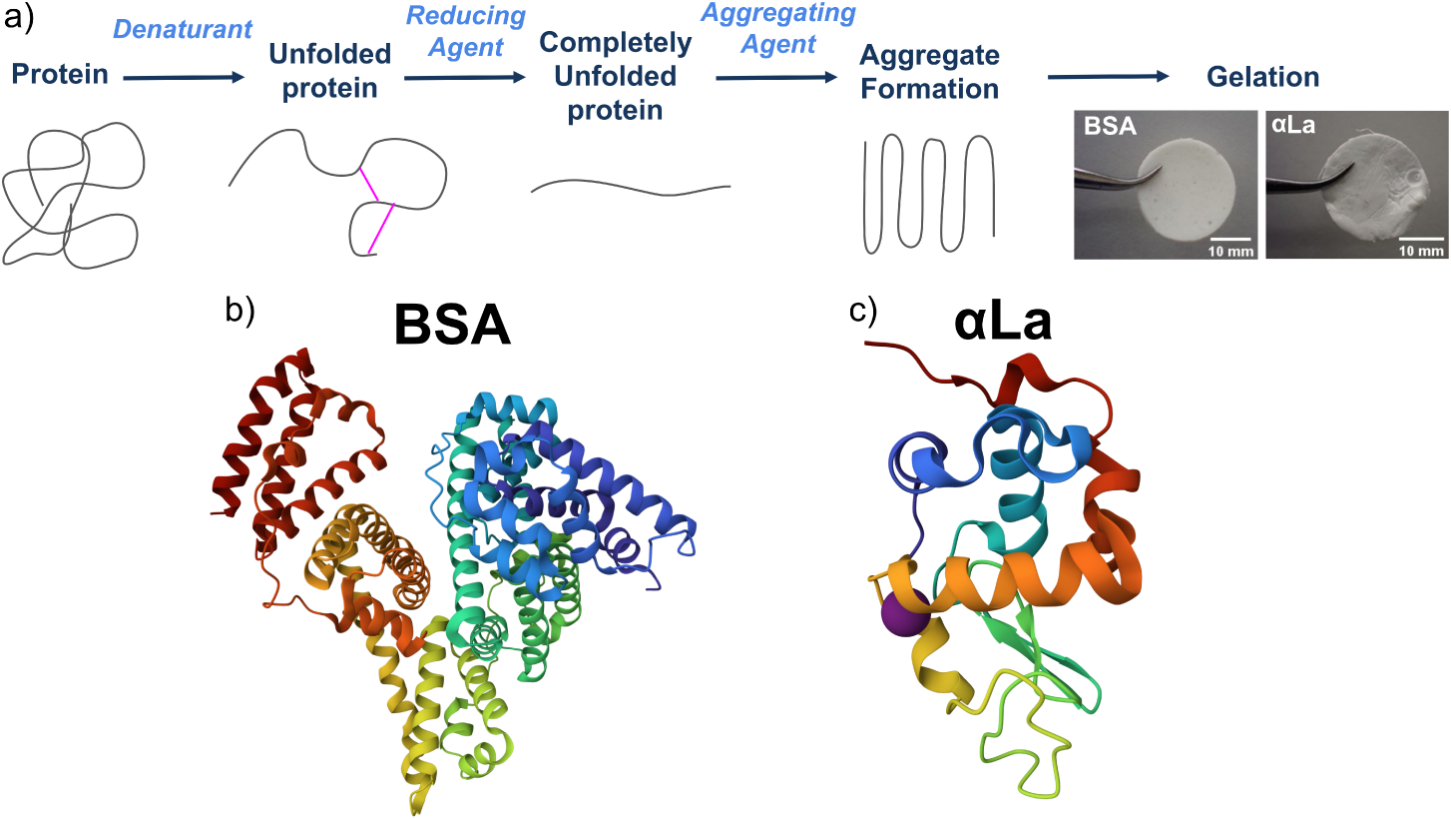
Schematic of protein-based underwater adhesive pathway involving commercial protein systems. (a) The protein-based underwater adhesive is a two-component system made up of a protein solution and a three-part initiator (denaturant, reducing agent, and aggregating agent). When the protein solution is mixed 1:1 with initiator and cured underwater, a protein gel is formed. Example protein gels can be seen here cured underwater in ASW for 24h, 80% w/v BSA (top) and 100% w/v αLa (bottom). (b, c) protein structure of BSA (b) and αLa (c), based on crystallographic structures 3V03 ^42^ and 1F6S^43^ in the Protein Data Bank (PDB)^44^ and visualized using Mol*.^45^ The bound Ca^2+^ ion in holo-αLa (c) is shown in purple.

We previously observed that our protein-based adhesives can perform substantially better in artificial seawater (ASW) than in deionized water,^6^ suggesting that ion content plays a large role in tuning adhesion. Salts can stabilize protein structure in aqueous solution, but in sufficient quantities will destabilize intramolecular forces and the solvation shell, “salting out” and aggregating the protein.^31^ Because of atomic factors like valency and size, all salts do not perform equally. While the Hofmeister series can describe general trends in ion impact on protein stability,^32^ environmental and protein-specific factors can also cause considerable variations in the ordering of ions between systems.^33–39^ The addition of salt can alter the properties of a protein-based adhesive in several ways. Changes in the denaturation or stabilization of the protein can hasten or hinder the solidification process. Salts can screen charges, altering the interaction of proteins with each other during aggregation and with the substrate during cross-linking can enhance the mechanical strength of the hydrogel,^40^ with the potential downside of embrittlement. Lastly, multivalent cations can undergo complexation with certain residues like aspartic acid or glutamic acid, producing a separate mechanism of cross-linking.^41^

Here, we investigate the effect of the four main chloride salts in seawater, NaCl, KCl, CaCl_2_, and MgCl_2_, on the properties of two protein adhesives made from the agricultural byproduct proteins bovine serum albumin (BSA, Figure 1b and Table S1) and alpha-Lactalbumin (αLa, Figure 1c and Table S2). Through the underwater adhesive pathway discussed, BSA and αLa denature in an aqueous solution and are influenced to form stable, insoluble aggregates. We probe the effect of salts on the gelation process, the mechanical properties of the cured hydrogel materials, and the lap shear strength of the glues on different substrates with the aim of developing new routes to understand and control the relationship between protein aggregate structure and the resulting adhesion.

## 2 Methods and Materials

### 2.1 Materials

Reagent-grade BSA (>90%) was purchased from SeraCare. BiPRO α9000, αLa, was kindly provided by Agropur, Inc. This protein was used as is from the manufacturer with no further purification (97.8% of the dry material was protein, with 91.3% of that protein being αLa). Benzalkonium chloride (BAC) (50% solution in water), Silica gel (230–400 mesh size, pore size 60 Å), salts: NaCl, KCl, CaCl_2_ · 2H_2_O, MgCl_2_ · 6H_2_O, urea (ACS reagent, 99–100.5%), and Drierite indicating desiccant (4 mesh) were purchased from Sigma-Aldrich. l-cysteine (l-cys) (97%) was purchased from Sigma-Aldrich and (99%) from MP Biomedicals. Artificial Sea Water (ASW) was produced using Instant Ocean Sea Salt (Spectrum Brands, Inc.) and deionized water (DI H_2_O). Salinity was measured with a hydrometer and maintained between 30–34 ppt and specific gravity was maintained between 1.021 and 1.026 (unitless).

### 2.2 Differential Scanning Calorimetry (DSC)

Commercial protein solution (10% w/v) was made by end over end rotation at 10 rpm for 24 h in a 15 mL conical centrifuge tube. Denaturant and salt solution were made by dissolving the solute into DI H_2_O at the specified concentration. 30–35 μL of each protein and denaturant solution was added (total 60–70 μL) in a 1:1 ratio to a TA Instruments high volume DSC pan. The aluminum pans were sealed using an O-ring on a TZero sample press with a high volume die set. A pan of ultrapure water was used as a reference for the protein solutions. Experiments were performed on a Discovery Series DSC (TA Instruments). Samples were equilibrated at 10 °C and held isothermally for 1 min. The pans were then ramped to 100 °C at a rate of 10 °C/min. The temperature at which the minimum heat flow occurred was recorded as the melting temperature of the protein. For protein samples without denaturants (protein in DI H_2_O), the 10% w/v protein solution was diluted with DI H_2_O to match the concentration of other sample mixtures. Each condition was measured a minimum of 3 times.

### 2.3 Adhesive Components (Protein and Initiator) Preparation

Based upon previous work establishing a working formula for BSA adhesives, the two-component system was extended to an additional commercial protein, αLa.^6^ Adhesive samples were generally made in a 1:1 ratio, by mixing a protein component (either 80% w/v BSA or 100% w/v αLa in DI H_2_O) with the corresponding initiator (BSA: 6 M urea, 1 M l-cys, 5% BAC or αLa: 8 M urea, 1 M l-cys, 7.5% BAC, dissolved either in DI H_2_O or the indicated salt solution). In some instances, the initiator also includes 30 wt % silica.

To make the protein solutions, dry BSA or αLa powder was dissolved in DI H_2_O (80% w/v or 100% w/v, respectively) in a sealed plastic container or conical centrifuge tube on a platform rotator (Fisher Scientific) at 10 rpm for at least 24 h at room temperature until the mixture was homogeneous. To make initiators, urea and l-cys were weighed into a beaker and BAC (50% solution in water) added. When needed, a specified amount of selected salt was added into the dry initiator components. All components were dissolved in DI H_2_O to the proper working concentration (BSA: 6 M urea, 1 M l-cys, 5% BAC or αLa: 8 M urea, 1 M l-cys, 7.5% BAC). The solution was heated to 60 °C with magnetic stirring until the components were fully dissolved. Once dissolved, silica was added if needed, and the solution was cooled to room temperature with continued mixing. Protein and initiator solutions were stored separately and kept at 4 °C until use. Adhesive samples were formed by mixing equal parts by mass protein solution and initiator.

### 2.4 Oscillatory Shear Rheology Sample Preparation and Testing

#### 2.4.1 Gelation Kinetics Testing

The gelation kinetics of samples were investigated using a Discovery HR-2 hybrid rheometer (TA Instruments) with a stainless-steel Peltier plate bottom stage and 25 mm diameter stainless-steel upper plate mounted in a parallel plate geometry. Immediately before testing, equal parts BSA protein solution and initiator were mixed by hand for 45 s before applying to the bottom plate. The gap between plates was set to 500 μm and excess adhesive was scraped from the sides of the plates. The sample was sheared at 0.1% strain and 1 Hz to obtain the storage modulus (*G*′), loss modulus (G″) and complex viscosity as the sample cured. The experiment was halted 10 points after a visible crossover (*G*′ > *G*″) occurred. Stage and plates were cleaned with DI H_2_O between measurements. Delay between mixing and data collection was recorded and accounted for in crossover time calculations, and each experiment was run in triplicate.

#### 2.4.2 Postcure Gel Moduli Testing

Adhesive samples were formed by mixing equal parts by mass protein solution and initiator. For BSA protein gels, samples were mixed by hand for 45 s before applying to clean and dry polyvinyl chloride (PVC) plates. Zip ties were used as spacers (1.13 mm) in between the PVC plates to ensure uniform thickness of the sample. A second PVC plate covered the adhesive and the sample was submerged underwater in the specified solution. Two 150 g weights were placed on top of the sample for 1 h while submerged. For α-La protein gels, the protein solution and initiator were mixed in a capped 10 mL syringe. Upon mixing, the syringe was assembled and adhesive was deployed underwater onto sheets of polytetrafluoroethylene (PTFE), again using zip ties as spacers. A second piece of PTFE was placed on top of the sample and a 200 g PTFE block was placed on top of the sample. After 1 h, the weights and the top plate were removed and the sample remained submerged for an additional 18–24 h. After that time, the large adhesive samples (Figure S1) were removed from the PVC or PTFE and cut into smaller disks using a 15/16” circular punch. Cut samples were stored in solution until rheological testing occurred, after 2 or 8 days of aging. Rheometry experiments were performed using a Discovery HR-2 hybrid rheometer (TA Instruments) using Trios software. The lower surface was a steel Peltier plate and the upper surface was a 25 mm diameter steel plate, configured in the parallel plate geometry. Surfaces were cleaned with DI H_2_O before and between measurements. Samples were loaded to a 50 N compressive load, then presheared for 30 s at 0.1 rads/s followed by a 60 s equilibration period prior to testing. Strain was varied from 10^–6^ to 10^–1^ at a frequency of 1 Hz to determine the storage modulus and loss modulus of each sample (Figure S2). The storage modulus was determined using the average of the linear portion of the storage modulus curve. Samples were run in triplicate.

### 2.5 Water Content

Adhesive samples were prepared using the same procedure as for the cured rheometry samples above. After 8 days of curing in solution, the hydrated gel disks were removed from the bath and the initial weight of the samples was measured. The disks were then placed into a desiccator with indicating Drierite desiccant for 1 week then subsequently in a drying oven at 60 °C for an additional week. After drying, the final mass of each sample was recorded and the water content of the sample was determined by the difference between original and final masses. Each condition was investigated with a minimum of three samples.

### 2.6 Tensile Testing

For mechanical characterization of adhesives samples were prepared according to ASTM D1708. Adhesive gel large disks were created using the same procedure as for cured rheometry samples above. The sample was submerged underwater in solution in a sealed container to avoid evaporation of solution and cured for 8 days. Flattened adhesive samples were peeled from the PVC sheet and punched into shape using a punch conforming to ASTM D1708. The cut specimen was 38.0 mm in length, 15.0 mm in width, and 1.13 mm thick, with the initial gauge length (L_0_) measured to be 22.0 mm in length and 5.0 mm in width. Gauge thickness was measured for each individual specimen. The tensile testing was performed using an Instron 68SC-05 (500 N load cell) with the sample clamped at both ends. Ten to 20 specimens were tested per adhesive mixture, at a loading rate of 5 mm/min to failure. Maximum force before fracture was used to calculate the ultimate tensile strength of the gel. αLa gels were too brittle to be cut into the proper shape, and thus αLa samples were not tested.

### 2.7 Raman Spectroscopy

Raman spectroscopy was performed on hydrated protein gel discs on glass slides prepared using the same method as for cured rheometry and water content samples. Spectra were acquired with a Renishaw inVia Raman upright microscope (Gloucestershire, United Kingdom) using a 785 nm Diode laser with 1200 lines/mm grating and a Peltier-cooled CCD detector. The laser was used at a power of 188 mW (resulting in 21 mW at the sample) and focused with a 50×/0.5 long working distance objective. Spectra were acquired from 280 to 1452 cm^–1^ with 1.5 s integration time and 128 accumulated scans. While the two scans had regions of overlap, the spectral ranges were selected in order to cover the entire desired range (280–1800 cm^–1^) while also ensuring each spectrum contained the required peak for normalization. Data were processed and analyzed using WiRE 4.1 software (Renishaw). Processing consisted of cosmic ray removal, background correction, and Savitsky–Golay smoothing (second order, 7 points). Spectra were normalized to the Raman peak at 1005 cm^–1^, corresponding to the C–C breathing mode in the phenylalanine amino acid that is commonly used for normalization of protein Raman spectra.^46^ Control samples consisting of a blank glass slide were also collected and analyzed for comparison. One spectrum was acquired per condition.

### 2.8 Lap Shear Sample Preparation

Lap shear substrates of aluminum (thickness: 0.125 in.), ABS (acrylonitrile butadiene styrene, thickness: 0.125 in.), and PC (polycarbonate, thickness: 0.177 in.) were custom manufactured (SendCutSend) to a dimension of 2.5” long and 0.5” wide. Prior to use, each substrate was sanded with 300 grit sandpaper (McMaster Carr) using a mouse sander (Black and Decker) and cleaned with ethanol and DI H_2_O before use.

Adhesive components were prepared as stated above in Section 2.3. Protein solutions and initiators were brought to room temperature and weighed out with a 1:1 ratio by mass, and protein + initiator were mixed by hand until well blended (∼30–45 s). The BSA adhesive was applied to one substrate in the air then bonded in a single lap joint geometry to another substrate submerged in the specified solution (DI H_2_O, ASW, or salt solution).^6^ For αLa, the adhesive mixture was transferred to a 20 mL Luer Lock syringe with 16 G needle for deployment. The αLa adhesive was applied to one substrate underwater via syringe delivery, then bonded in a single lap joint geometry to another substrate while still submerged in the specified solution.

For both BSA and αLa, the samples were aligned to an overlap of 0.5 in using a custom 3D printed mold (Figure S3a) with a 0.015 in (0.381 mm) thick gap between the samples, and a 250 g weight was placed on the sample joint (Figure S3b). The molds were coated with silicone vacuum grease before use to allow for easy release of the samples. Samples were removed from the mold after 24 h and continued conditioning for a total of 1 week in their respective solution conditions. Excess glue was removed from all sides of the lap shear sample with a plastic razor blade. Long-term (4, 8, and 12 weeks) lap shear samples were made similarly. Upon removal from the molds after 1 week, the samples were left in their curing solution for up to 12 weeks undisturbed before testing.

### 2.9 Lap Shear Sample Testing

Lap shear samples were tested according to ASTM D1002.^47^ The lap shear samples were designed with holes on each side and mounted on the tensile tester using Clevis pins. Samples were tested using either an Instron 68SC-05 (500 N load cell) at the US Naval Research Laboratory or an ADMET eXpert 7601-Q (500 N load cell) at the US Naval Academy (Figure S3c). The bond area was assumed to be 161 mm^2^, based on the designed sample width and overlap area of the samples in the mold. Lap shear samples were pulled apart at a rate of 1.5 mm/min (estimated as ∼0.07 s^–1^ shear strain rate) until break. The maximum force of the adhesive at the break was recorded and the failure mode was noted. Samples were pulled in sets of 3 or 6 to determine statistical relevance.

### 2.10 Data Analysis

Data was primarily processed and analyzed using home-built Python routines. All data are reported as mean ± standard error of the mean to reflect the precision of the measurements rather than variability within the sample. Error bars indicate the standard error of the mean for each data set. Comparative statistics were performed using one way ANOVA tests, while correlations were assessed through linear regression.

## 3 Results

### 3.1 Cations Alter the Stability of Native Protein Structures

The Hofmeister series describes the ability of ions to influence the structure and stability of proteins through the modulation of water structure and electrostatic interactions (Figure 2a). The Hofmeister series has been well characterized across various systems, but the relative ordering of ion denaturation power is known to depend on the protein, ion concentration, and other environmental factors.^33,48,49^ Thus, we began by investigating the relative effect of each of the common seawater cations on the stability of the native protein structure under conditions relevant to our adhesives. We measured the thermal denaturation of aqueous Bovine Serum Albumin (BSA) and α-Lactalbumin (αLa) solutions containing the four most abundant seawater salts, NaCl, KCl, MgCl_2_, and CaC_2_, using Differential Scanning Calorimetry (DSC), a powerful analytical technique for studying thermodynamic transitions in biological systems.^50–52^

**Figure 2:**
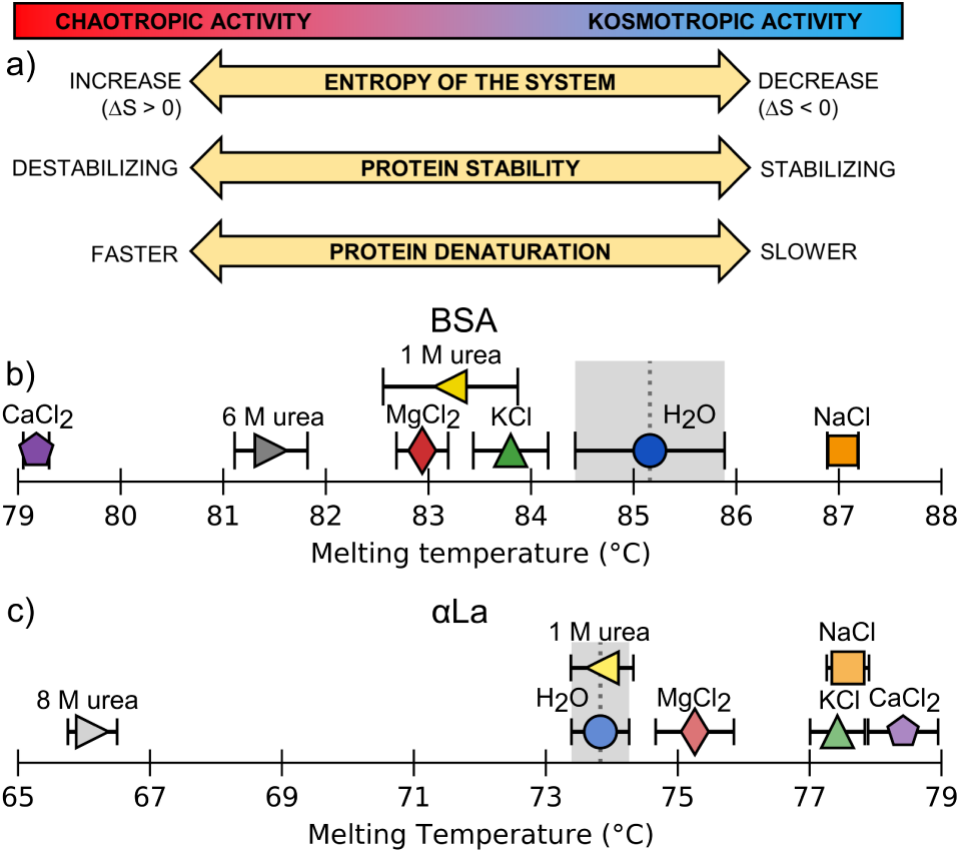
Chao-/Kosmo-tropic activity and measured denaturation temperatures for the BSA and αLa protein systems. (a) Schematic illustration of the Hofmeister series and distinct properties between chao/kosmotropes^48,49^ (b, c) Melting (unfolding) temperatures of 5% (w/v) BSA (b) or αLa (c) in DI H_2_O (blue circles) or mixed 1:1 with 1 M denaturant (KCl, green upward triangles; NaCl, orange squares; MgCl_2_, red diamonds; CaCl_2_, purple pentagons; or urea, yellow left facing triangles). Also shown is protein mixed with 6 M urea (b) or 8 M urea (c), (gray right facing triangles). *n* = 3–8; error bars indicate the standard error of the mean.

Heating a protein to a certain temperature allows the protein to unfold from its native state into a denatured conformation. This transition manifests as a local change in the heat capacity of the sample, the midpoint of which is defined as the melting temperature of the protein (*T*_m_). A higher melting temperature indicates a protein with higher thermal stability.^34,53^ We measured the melting temperature of 5% w/v solutions of BSA in DI H_2_O to be 85.2 ± 0.7 °C (Figure 2b), notably higher than the commonly reported literature value of 60 – 70 °C,^54–56^ possibly due to crowding effects in the highly concentrated protein solution stabilizing the native structure. When 10% w/v solutions of protein were mixed 1:1 with a 1 M NaCl solution (forming a 5% w/v, 0.5 M mixture), the *T*_m_ increased slightly to 87.0 ± 0.2 °C, while when mixed with 1 M KCl, MgCl_2_, and CaCl_2_ the *T*_m_ drops to 83.8 ± 0.4 °C, 82.9 ± 0.3 °C, and 79.2 ± 0.1 °C, respectively. In comparison, mixing with a 1 M solution of urea has a small effect on the melting temperature (*T*_m_ = 83.2 ± 0.7 °C), while a 6 M urea solution more dramatically decreases the melting temperature to 81.5 ± 0.4 °C.

While the thermal denaturation of BSA largely shifts to lower temperatures with the addition of salt, the melting temperature of αLa is increased by the four salts studied here. α-Lactalbumin, a smaller protein with a noted calcium binding site,^57^ had melting temperature in DI H_2_O of 73.8 ± 0.4 °C.^58^ The addition of 1 M MgCl_2_ slightly increased the *T*_m_ to 75.3 ± 0.6 °C, followed by larger increases with KCl and NaCl to 77.4 ± 0.4 °C and 77.6 ± 0.3 °C, respectively. αLa mixed with 1 M CaCl_2_ gave the largest increase in the melting temperature, with *T*_m_ = 78.4 ± 0.5 °C. 1 M Urea had a negligible effect on the thermal stability of αLa (*T*_m_ = 73.9 ± 0.5 °C), while the stability was significantly perturbed by the addition of an 8 M urea solution, decreasing to 66.1 ± 0.4 °C. These results confirm that the ocean salts studied can alter the stability of native BSA and αLa protein structures, with strengths that can rival or surpass the commonly used denaturant urea at comparable concentrations.

### 3.2 Increasing Ionic Strength Lowers Viscosity and Alters Curing Kinetics of Adhesives

Protein adhesive gels “cure” through a denaturation and aggregation process. First, the denaturants in the initiator work to disrupt the protein secondary and tertiary structure, forcing the proteins into partially unfolded or molten globular states.^6^ In this state, the protein is no longer stable in solution and will begin to form insoluble aggregates. As the aggregates grow, the solution becomes more viscous and eventually forms a solid gel. The kinetics of this process are crucial to forming a successful underwater adhesive—if the adhesive cures too slowly the miscible protein precursors will dissolve away before the bond is formed. Increased viscosity is also helpful for bonding, keeping the adhesive in place when depositing and hindering material loss.^22^

We sought to alter the mixture properties during the cure process by adding salts into the initiator solution in addition to the denaturants already present (urea, l-cys, and BAC). Hereafter we refer to the salt concentration included in the initiator, but as the adhesive is a 1:1 mixture of protein and initiator the actual salt concentrated is halved. The inclusion of salt in the initiator may affect curing and viscosity through several mechanisms. First, the changes in protein thermal stability seen in Figure 2 may accelerate or delay the denaturation of proteins. Once denatured, increased ionic strength will suppress electrostatic repulsion, which could increase aggregation, and divalent ions may lead to complexation or bridging between proteins or aggregates.^40,59^ Decreased intermolecular forces may lower the viscosity of the mixture, while increased aggregation may make viscosity rise at a faster rate.

We investigated the effects of ocean salts on the postmix viscosity and curing kinetics of adhesives by monitoring changes to the physical properties of the adhesive mixture during curing through oscillatory shear rheology. We first study the complex viscosity of the solution, choosing a time point of 90 s after mixing to minimize aggregation and curing effects while still allowing for practical sample preparation (Figure 3a). The addition of 0.01 M salt into the initiator decreased the viscosity of adhesive mixtures 90 s after mixing from a reference value of 27.0 ± 0.3 Pa·s without salt in the initiator (Figure 3b), regardless of the identity of the salt. Further decreases were seen when salt concentration was increased to 0.1 and 1 M, with the viscosity adhesives with 1 M NaCl and 1 M KCl, initiators decreasing over an order of magnitude from the DI H_2_O reference. A notable exception is 1 M CaCl_2_, which saw a dramatic increase in viscosity, rising more than an order of magnitude from 0.1 to 1 M CaCl_2_.

**Figure 3:**
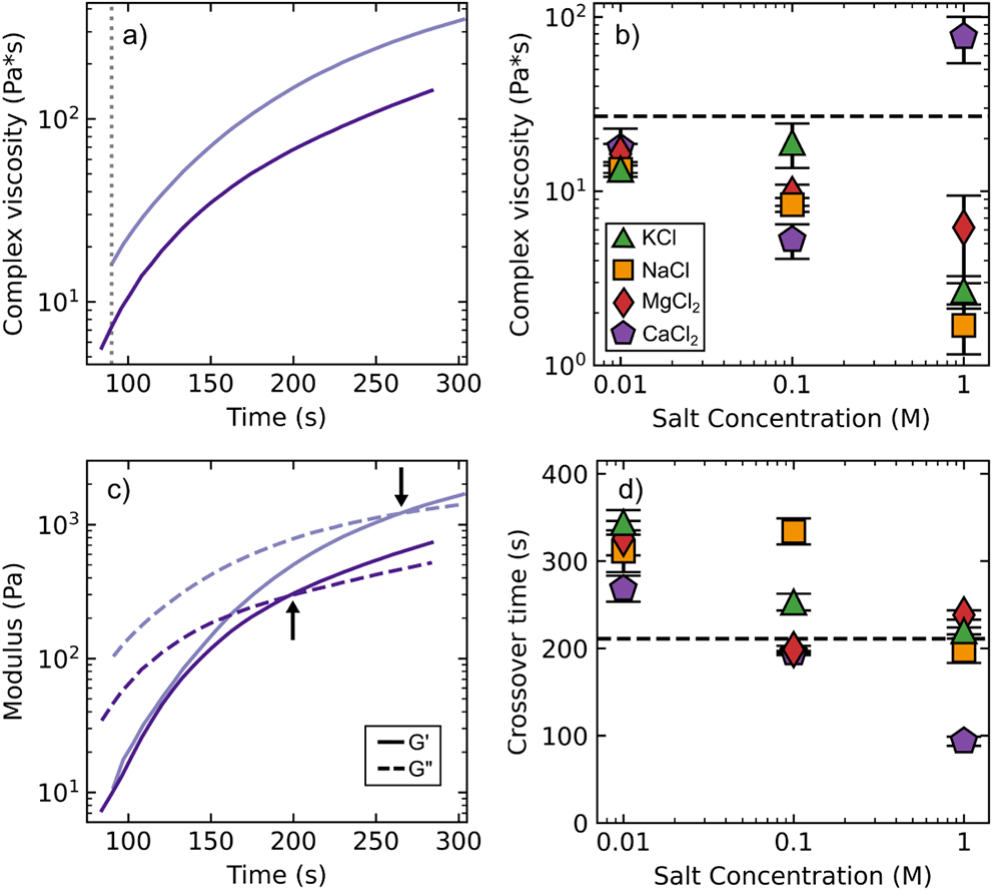
Characterization of the effect of viscosity and crossover time for gel formation with BSA protein. (a) Complex viscosity of BSA adhesives with 0.01 M CaCl_2_ (upper) and 0.1 M CaCl_2_ (lower) over the course of the gelation process. The viscosity values at 90 s (dashed line) were selected for comparison between conditions. (b) Viscosity 90 s after mixing for BSA adhesives with different concentrations of salts: KCl (green triangles), NaCl (orange squares), MgCl_2_ (red diamonds), and CaCl_2_ (purple pentagons). The dashed line indicates the viscosity of adhesives without salt (DI H_2_O initiator). (c) Storage modulus (*G*′, solid lines) and loss modulus (*G*″, dashed lines) over the gelation process for the same adhesive mixtures as in part a). Crossover time, where *G*′ = *G*″, is indicated by the arrows. (d) Crossover time of BSA adhesives over a range of concentrations of KCl (green triangles), NaCl (orange squares), MgCl_2_ (red diamonds), and CaCl_2_ (purple pentagons). The dashed line indicates the crossover time of adhesives without salt (DI H_2_O initiator). Curves in panels a and c are representative; points in panels b and d are averages with *n* = 3. Error bars indicate the standard error of the mean.

The curing kinetics of BSA adhesives were monitored through tracking the ratio of the storage modulus (*G*′, elastic nature of the material) and loss modulus (*G*″, dissipative nature of the material) over time. Immediately after mixing, *G*″ > *G*′, indicative of a liquid mixture (Figure 3c). Over time, *G*′ rises at a faster rate than *G*″ as the material begins to solidify. The point where G′ = *G*″, the crossover point, is commonly used as a measure of the gelation time of a material.^60^

We measured the crossover time of BSA adhesive mixtures with a range of salt concentrations in the initiators. With no salt in the initiator, the crossover time for BSA mixed with a 6 M urea, 1 M l-cys, and 5% BAC initiator is 211 ± 1 s (Figure 3d). The addition of any of the salts at 0.01 M increased the crossover time from this reference value, indicating that gelation occurred more slowly. However, further increases in salt concentration led to decreases in the crossover time, with 1 M salts (except CaCl_2_) having approximately equal crossover times to BSA gels without salt. While in general mixtures with lower viscosities at 90 s had lower crossover times, CaCl_2_ in particular dramatically accelerated gelation. It is likely that the anomalous viscosity increase for 1.0 M CaCl_2_ at 90 s is the result of the mixture nearing its crossover point, while the other mixtures still had *G*″ ≫ *G*′. The addition of salt is thus capable of tuning the gelation kinetics, either slowing down cure at low salt or speeding up cure at high salt concentrations. αLa adhesives cure through a more complex mechanism than BSA and will be the subject of a future investigation.

### 3.3 Moderate Ion Content Enhances the Mechanical Properties of the Bulk Gel

BSA and αLa adhesive gels continue to cure and harden over time as they are soaked in water. Prolonged immersion in ocean salt solutions may lead to further swelling of the gels or encourage the rearrangement of proteins to form more intermolecular bridges or adopt tougher, lower energy conformations. We first characterized the water content of adhesive gels that contained salt in the initiator and underwent 8 days of immersion in salt solution. For BSA gels, the water content decreased slightly from 70.1 ± 0.5% to 64.4 ± 0.2% when aged in artificial seawater (ASW) instead of DI water, suggesting that the salinity of the bath does alter the water content of the final gel (Figure 4a). When adhesives were made with and soaked in 0.1 M salts, water content only minorly decreased compared to the DI H_2_O control. However, 1 M salt solutions saw more marked changes, with 1 M MgCl_2_ and CaCl_2_ solutions decreasing the water content of gels to 60 ± 1% and 58.7 ± 0.4%, respectively. αLa adhesives showed similar trends, with minor increases in water content in 0.1 M salt solutions and strong decreases in water content for 1 M divalent salt solutions (Figure 4b).

**Figure 4:**
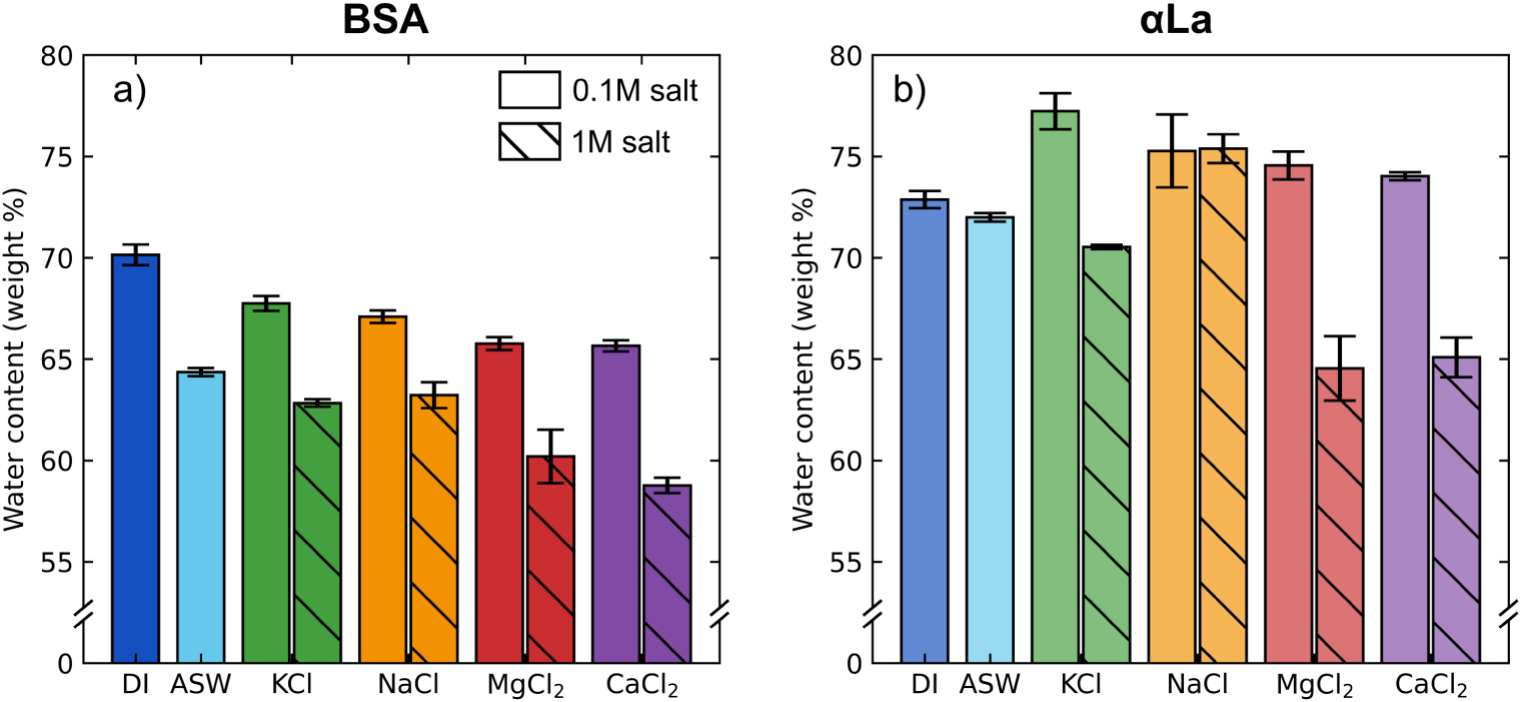
Water content of protein adhesive gels after curing. Mass fraction of water in BSA (a) and αLa (b) gels after 8 days curing in the specified solution. For all conditions *n* ≥ 3 and error bars indicate the standard error of the mean.

Adhesive performance is highly sensitive to the physical properties of the bulk material. We therefore evaluated the effect of salt content on the storage modulus (*G*′) of cured adhesive gels. In agreement with our prior studies, the storage modulus of BSA generally increased from 2 days to 8 days of aging, indicating continual rearrangement of proteins and stiffening of gels (Figure S4a–c). To separate the role of aging adhesives in salt solutions from the effect of salt-containing initiators, we compared the storage moduli of gels made with salt-free initiators and aged in 0.1 M salt solution (S condition) to those both made with 0.1 M salt in the initiator and aged in 0.1 M salt solution (I/S condition). Aging BSA adhesives in salt alone does not significantly alter the storage moduli of the gel (Figure 5a), but including salt in the initiator leads to larger changes. 0.1 M CaCl_2_, regardless of condition, softens the final BSA adhesives. αLa adhesives have higher storage moduli than their BSA counterparts (Figure 5b *p* < 0.001) but have higher variability in the storage modulus after curing, likely due to their more brittle nature leading to damage during sample preparation. Inclusion of divalent salts in the initiator appears to decrease the shear modulus of αLa gels after 2 days (Figure S4d–f), but this discrepancy disappears after 8 days of aging and there are no longer significant differences between the S and I/S conditions. 0.1 M CaCl_2_ leads to moderately stiffer αLa gels than 0.1 M KCl, opposite of what was observed for BSA.

**Figure 5:**
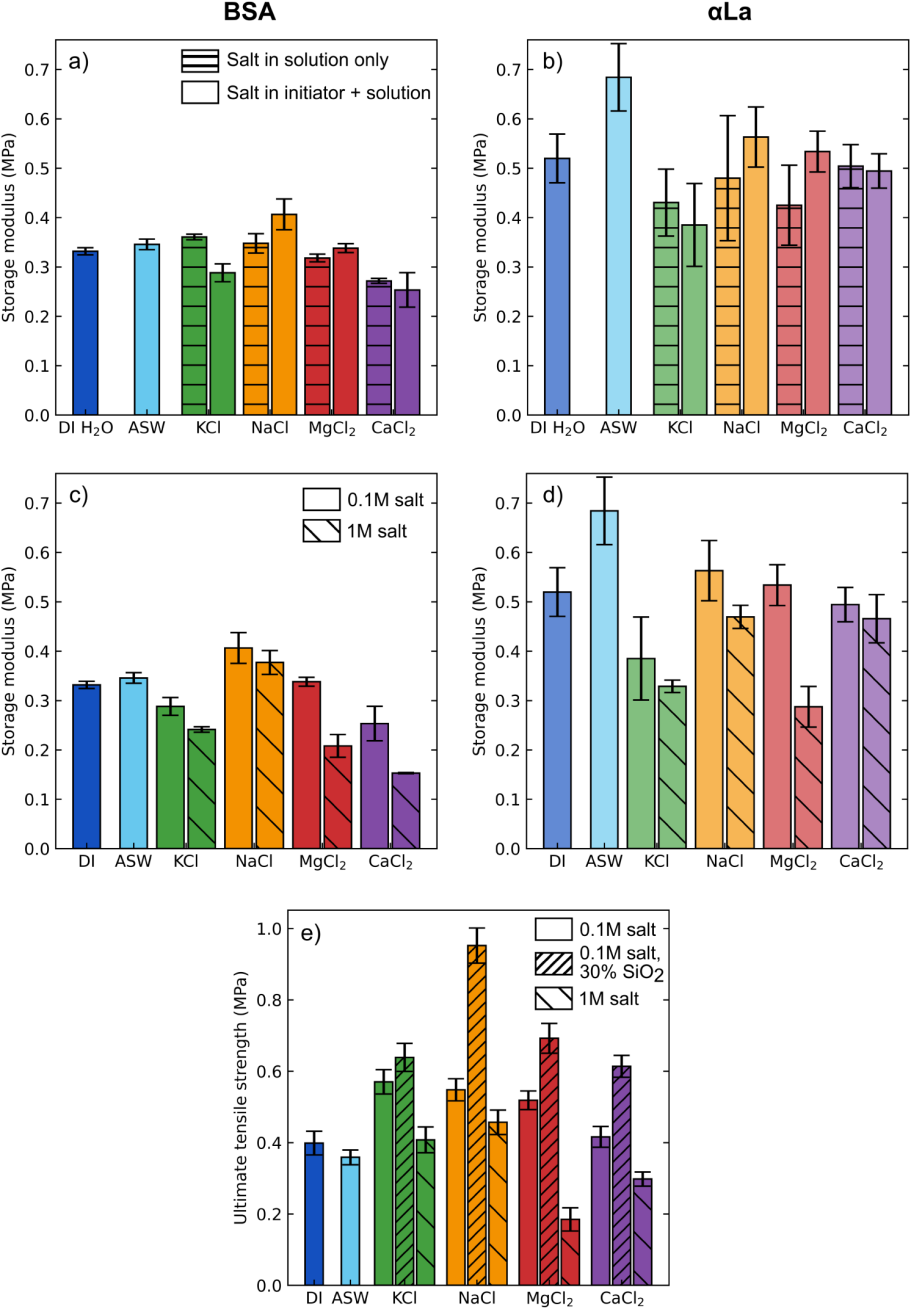
Bulk mechanical properties of protein adhesive gels after curing. (a, b) Storage moduli of BSA (a) and αLa (b) gels after 8 days of curing in 0.1 M salt solutions. Hatched bars had initiators made with DI H_2_O, while plain bars had initiators made with the corresponding salt solution. (c, d) Storage moduli of BSA (c) and αLa (d) gels made with initiators containing 0.1 or 1 M salt and cured in the corresponding salt solution for 8 days. (e) Ultimate tensile strength of 8 day cured BSA gels with salt in both initiator and aging solution as tested through ASTM D1708. For panels a–d, *n* = 3 while for panel e *n* ≥ 4. Error bars indicate the standard error of the mean for all plots.

As the salt concentration is increased to 1 M, larger effects are seen on the stiffness of protein gels (Figure 5c). Apart from NaCl, the lone stabilizing salt, BSA gels are notably softer in 1 M salt solution than in 0.1 M salt, thus increasing salt concentration appears to be detrimental for the final storage modulus of the gel (*p* < 0.05). αLa adhesives, on the other hand, show a strong increase in storage moduli when aging in ASW compared to DI H_2_O (0.68 ± 0.07 MPa vs 0.52 ± 0.05 MPa, respectively, Figure 5d). Except for MgCl_2_, storage moduli of αLa gels had minor or insignificant decreases in the storage modulus when salt concentration increased from 0.1 to 1 M, but all storage moduli were significantly lower than those of samples aged in ASW (*p* < 0.01).

The adhesion of protein gels depends upon fracture strength of both interfacial bonds and the bulk gel network. We sought to characterize the changes to bulk fracture strength of cured BSA gels through tensile testing in order to better decouple changes in adhesion due to bulk and interfacial phenomena. The inclusion of 0.1 M salt in gels (in both initiator and solution) boosted the ultimate tensile strength, the maximum stress sustained before failure, of BSA gels by up to 43% in comparison to DI H_2_O samples (Figure 5e). On the other hand, the use of 1.0 M salts tended to decrease the ultimate tensile strength of gels (*p* < 0.001). The inclusion of silica fillers into the adhesive has been previously shown to increase the storage modulus and bond strength of protein gels,^6^ and thus we explored the effect of the addition of 15% silica into gels in the presence of 0.1 M salt. Adding silica universally increases the ultimate tensile strength of protein gels (*p* < 0.001) while decreasing the strain at failure (Figure S5¸ *p* < 0.001), suggesting that the resulting material is stiffer and more brittle. NaCl gels were particularly affected by silica, possibly due to the salt encouraging protein interactions with the hydrophilic silica particles. These experiments indicate that the identity and concentration of salt in adhesives can strongly alter the mechanical properties of the cured gels, with strong implications for the final performance as an adhesive.

### 3.4 Raman Spectroscopy Reveals Changes in Protein Aggregate Secondary Structure

Mechanical tests are informative about the bulk properties of the adhesive gels, yet do not provide direct insight into microscopic changes within the adhesives. Raman spectroscopy can provide insight into the conformation of proteins, revealing the effect of salt on protein folding and self-assembly. In particular, spectral bands between 500–550 cm^–1^ can be attributed to disulfide bonds in the protein network, the only source of covalent intermolecular bonds within the gel.^61^ αLa gels exhibit a strong Raman peak at ∼500 cm^–1^ (Figure 6), consistent with the high concentration of cysteine residues within the protein. A weaker peak at ∼515 cm^–1^ is also visible, likely due to disulfide bonds in a different conformation.^61,62^

**Figure 6:**
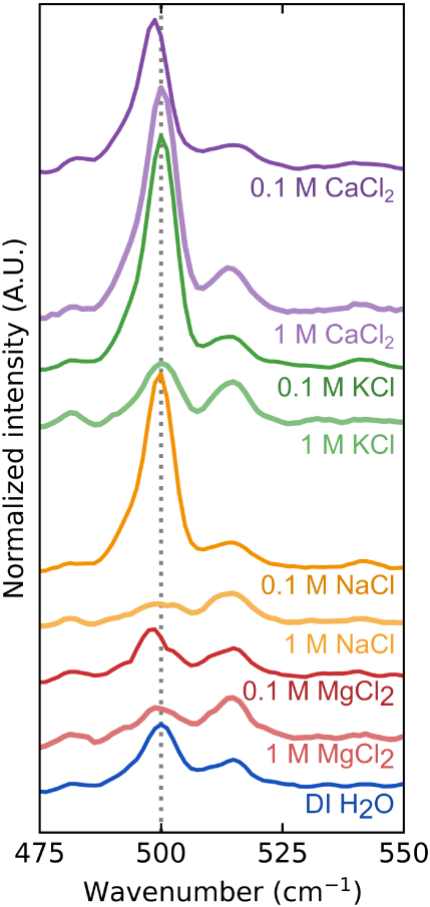
Structural analysis of protein adhesives through Raman spectroscopy. Raman intensity in the disulfide bond region of αLa gels exposed to the following conditions from bottom to top: DI H_2_O (dark blue), 1 M MgCl_2_ (light red), 0.1 M MgCl_2_ (dark red), 1 M NaCl (light orange), 0.1 M NaCl (dark orange), 1 M KCl (light green), 0.1 M KCl (dark green), 1 M CaCl_2_ (light purple), and 0.1 M CaCl_2_ (dark purple). All spectra are normalized to the peak intensity at 1005 cm^–1^, attributed to C–C vibrations in phenylalanine residues, and spectra are shifted vertically for clarity. Complete spectra are available in Figure S6.

The intensity of the 500 cm^–1^ peak increases sharply in 0.1 M KCl, NaCl, and CaCl_2_ but is unaffected in 0.1 M MgCl_2_. At higher salt concentrations the intensity of this main peak slightly decreases from the DI H_2_O control, except in 1 M CaCl_2_, where the intensity continues to rise. At the same time, the weaker 515 cm^–1^ peak is not significantly altered by any 0.1 M salt but increases in prominence in 1 M salts. From this, we infer an increase in the number of disulfide bonds at moderate ionic strength followed by a decrease in disulfide bond content and increased prevalence of other disulfide bond conformations at higher ion concentration. However, we note that despite 1 week of soaking these samples in solution we cannot rule out the presence of residual l-cys from the initiator, possibly leading to increased disulfide signal that is unrelated to protein–protein cross-linking.

### 3.5 Lap Shear Strength of Salt Containing Protein Gels

We examined the lap shear strength of BSA and αLa adhesive gels deposited on polycarbonate (PC), acrylonitrile butadiene styrene (ABS), and aluminum substrates to understand adhesion to hydrophobic polymer and hydrophilic oxide surfaces. Our lap shear samples were prepared using a custom-made mold to fix the bond thickness at 0.015”. This prevents variations in viscosity from changing the thickness of the bondline but reduces adhesion overall. As these are underwater adhesives, at least one adherend in each sample was bonded underwater. We note that as the adhesive mixtures are roughly 50–60% water by mass, even application to dry surfaces involves bonding in the presence of water.

Lap shear strength of BSA adhesives in 0.1 M KCl was slightly enhanced on hydrophobic PC and ABS substrates in comparison to DI H_2_O and ASW controls, while bond strength was significantly improved with 0.1 M CaCl_2_ (*p* < 0.001). 0.1 M NaCl or MgCl_2_ did not substantially alter the lap shear strength of BSA on PC or ABS (Figure 7a-b). Lap shear on aluminum was highly variable, possibly due to challenges in reproducibly bonding samples on strongly hydrated substrates (Figure 7c). 0.1 M NaCl showed the strongest effect, nearly tripling the bond strength compared to samples in DI H_2_O. Increasing salt concentration to 1 M generally weakened lap shear strength (*p* < 0.001), leading to samples that broke apart during handling in many of the monovalent salt conditions. Notably, 1 M CaCl_2_ was less detrimental to bond strength, in spite of the previously measured decrease in storage modulus and tensile strength under these conditions. Despite the addition of silica increasing the tensile strength of the gels, it frequently decreased bond strength on both hydrophobic and hydrophilic substrates. The drop in lap shear strength is roughly in inverse relation to the increase in ultimate tensile strength with silica observed in Figure 4, hinting at a competition between cohesion and adhesion in the presence of silica. Failure of BSA gels generally occurred through adhesive failure, suggesting that we did not reach the limits of mechanical strength in the bulk gel before failure occurred.

**Figure 7:**
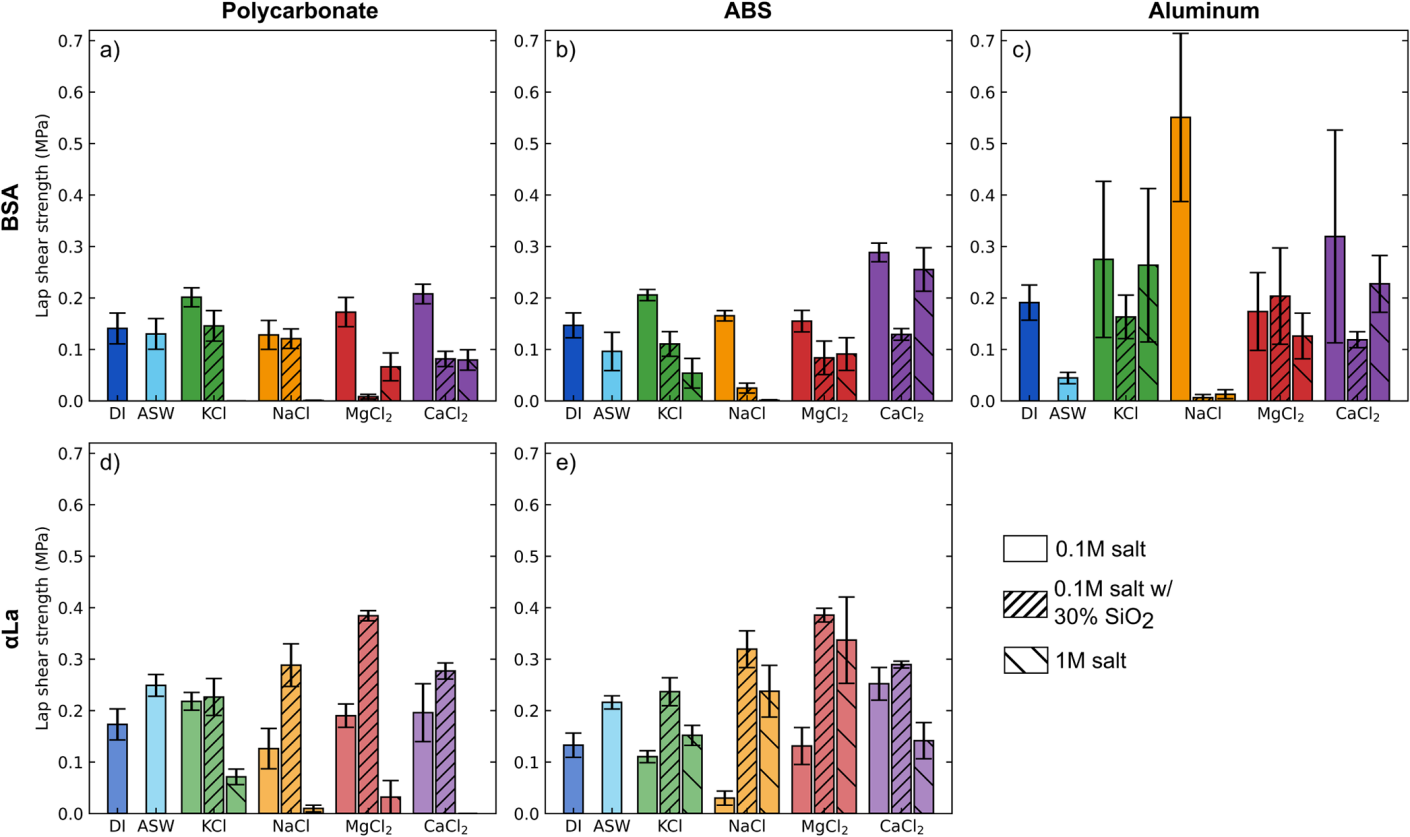
Lap shear strength of adhesives. BSA (a–c) and αLa (d, e) adhesives with polycarbonate substrates (a, d), ABS substrates (b, e), or aluminum substrates (c), after aging for 1 week in the specified solution. Both initiator and aging solution contained the specified salt, with the exception of ASW (salt only in the solution). For all conditions *n* ≥ 6 and error bars indicate the standard error of the mean.

αLa adhesives bind strongly onto hydrophobic surfaces, typically having higher lap shear strengths than their BSA counterparts (Figure 7d,e) but adhere poorly to aluminum. Nearly all αLa samples on aluminum failed adhesively before they could be tested, revealing extremely weak bond strengths to aluminum surfaces. Lap shear strength of αLa adhesives increased in ASW by 44% on PC and 63% on ABS in comparison to DI H_2_O, suggesting that the presence of ions can enhance adhesive performance. At lower salt concentrations (0.1 M), lap shear strength was largely unaffected on PC and ABS. 1 M salt solutions were highly detrimental for adhesive performance on PC (*p* < 0.001), but could enhance bond strength on ABS, particularly for NaCl and MgCl_2_ solutions. Interestingly, silica enhanced the lap shear strength of αLa adhesives (*p* < 0.01), yielding our highest recorded bond strengths on PC and ABS with 0.1 M MgCl_2_. At the same time, failure modes switch from adhesive without silica to cohesive with silica, indicating that the presence of silica may enhance interfacial interactions. These results suggest that the inclusion of salts can significantly alter and sometimes enhance the adhesion strength of protein adhesives, but that proteins that are stabilized by the presence of salts may see more benefit to the inclusion of salt in adhesives.

A major concern for underwater adhesives is their long-term durability upon immersion in water. Even strong adhesive joints can be susceptible to water uptake and interfacial displacement, leading to bond weakening or failure.^63^ We investigated the lap shear strength of both BSA and αLa adhesives on ABS substrates over 3 months of immersion in ASW. The lap shear strength of αLa adhesives continues to improve for 1 month of aging, while for BSA strength increases for up to 2 months in ASW (Figure 8). After this period, a decrease from the peak strength is seen for both samples, but bond strength remained comparable to the initial lap shear strength at 1 week. The decrease in bond strength was coincident with an increase in turbidity of the bath and development of an unpleasant odor, indicating that microbial growth in the stagnant water may be responsible for the observed drop in adhesion.

**Figure 8:**
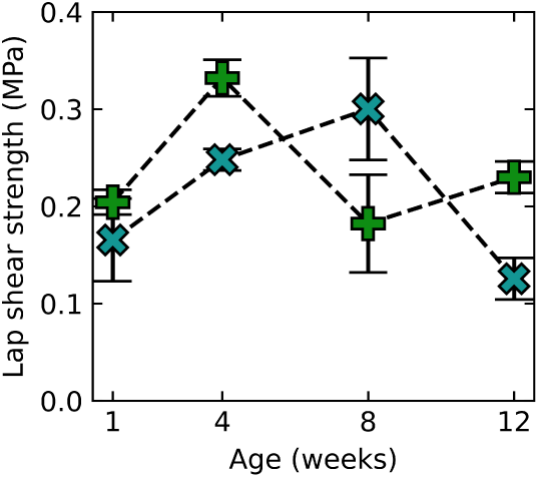
Lap shear strength of adhesives over time. Lap shear strength of BSA (blue x) and αLa (green plus) on ABS substrates over up to 12 weeks of ASW immersion. Each point represents an average of 3 samples and error bars indicate the standard error of the mean.

## 4 Discussion

The inclusion of salts in protein adhesive gels can be a simple approach to tune the intermolecular forces during curing, linking broad environmental effects to physical adhesive properties. Strong adhesives must both adhere to the surface and cohere within the material, goals that often are in conflict. We find that at similar ionic concentrations, there is a general decrease in bond strength with increasing storage modulus and tensile strength (Figures 9a,b and S7). Increasing the bulk strength of the material came at a cost to interfacial interactions, a known trade-off in adhesive design.^64^ On hydrophobic substrates, BSA adhesives generally shows small but significant increases in adhesion at 0.1 M salt concentrations as the salt enhanced interfacial interactions between the protein and substrate. More complete unfolding of the protein early in the curing process likely provides more area for hydrophobic or π-π stacking interactions between the protein and surface. Adhesion to aluminum, on the other hand, is strongly impacted by ion content. The presence of salts may help expose hydrogen bonding groups or charged residues within the protein, allowing for more interactions with the hydrophilic metal oxide. Cationic residues in particular have previously been shown to aid in interfacial water removal, and the exposure of BSA’s many cationic amino acids could allow it to bind more strongly to aluminum.^19,65^

**Figure 9:**
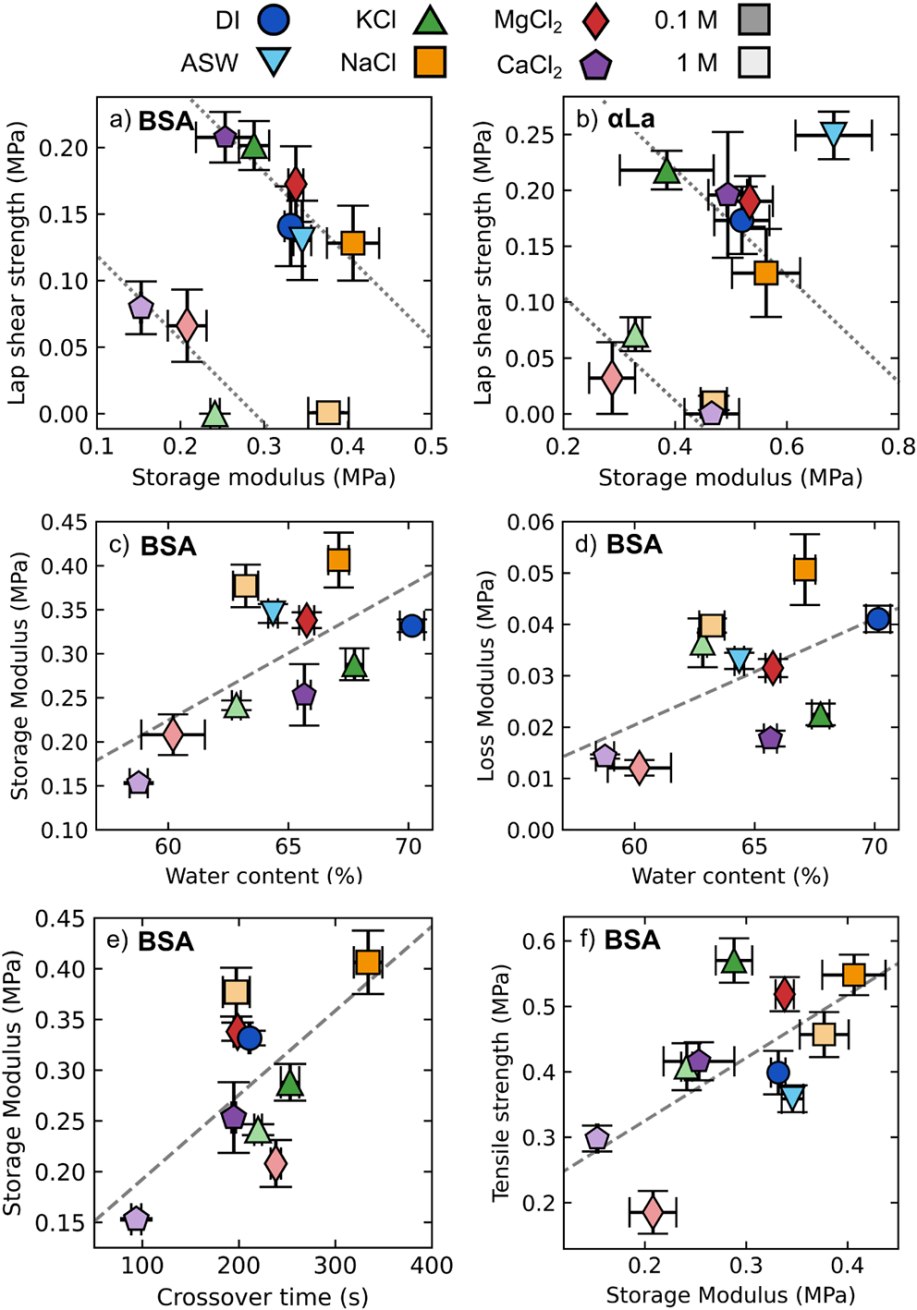
Trends in material properties. For all plots, DI H_2_O is shown in dark blue circles, ASW in light blue downward triangles, KCl in green upward triangles, NaCl in orange squares, MgCl_2_ in red diamonds, and CaCl_2_ in purple pentagons. 0.1 M salts are shown as darker colors, while 1 M salts are lighter colors. (a, b) Lap shear strength vs storage modulus of BSA (a) and αLa (b) adhesives deposited on PC adherends. Dotted lines in panels a and b are solely to guide the eye. (c, d) storage modulus (c) and loss modulus (d) vs water content of BSA gels. The trend lines indicate linear regressions with *p* = 0.04, *r* = 0.66 and *p* = 0.09, *r* = 0.56, respectfully. (e) 8-day storage modulus vs crossover time for BSA gels exposed to different salt conditions. The line indicates trend with *p* = 0.065 and *r* = 0.64. (f) Tensile strength vs storage modulus of BSA gels; line indicates trend with *p* = 0.040 and *r* = 0.65. Error bars indicate the standard error of the mean.

αLa-based adhesives, with their higher tolerance for high salt and lower affinity for oxide surfaces,^66^ fared better in ASW than in any 0.1 M salt concentrations, barring CaCl_2_. ASW solutions, at roughly 0.5 M total cation concentration (mostly Na^+^),^67^ could be close to an ideal concentration for balancing adhesive and cohesive interactions or could result in cooperative effects that enhance both bulk stiffness and adhesion. In contrast to the addition of 0.1 M salt, a surprising result is that 1 M salt conditions almost universally weakened both the adhesion and cohesion of both protein gels. While we did not seek to optimize salt concentration here, we hypothesize that BSA adhesives may exhibit higher storage moduli and bond strengths at lower ionic strengths (perhaps 0.01 M), while the mechanical properties of αLa gels may be maximized around 0.5 M salt concentration. Proteins that are highly salt tolerant or use salts to stabilize their native structure may be better candidates for adhesives in brackish or marine environments.

To rationalize the effects of salts on the strength of underwater–protein adhesives we investigate the relationships between material properties of the gels. We originally hypothesized that increased swelling leads to softer hydrogels (or vice versa) and weaker adhesion, as commonly reported in literature.^68–73^ Instead, both storage modulus (*p* = 0.04, *r* = 0.66, Figure 9c) and loss modulus (*p* = 0.09, *r* = 0.56, Figure 9d) rose with increasing water content, with lap shear strength also tending to increase for more swollen gels (Figure S8). Furthermore, αLa adhesives have both higher storage moduli and water content than BSA gels despite the αLa adhesives having a higher initial protein mass fraction. In our case, the change in water content does not follow the classic swelling equilibrium^74^ and these trends likely are the result of differences in network structure or the location of water within the material.^16^

To further shed light on the weaker strengths at high salt conditions, we examine the crossover time of the adhesives for clues in changes to protein aggregation kinetics. At low ion concentrations the addition of salt commonly enhances the stability of proteins (“salting-in”) by stabilizing the protein’s hydration shell and screening electrostatic charges that may otherwise favor aggregation. On the other hand, high salt concentrations disrupt the protein’s hydration shell and interfere with the hydrogen bonds that stabilize the protein, while the screening of electrostatic charges reduces repulsive forces between denatured proteins and decreases colloidal stability.^40,59^ Collectively, this results in the observed increase in crossover time at lower ionic strengths followed by the subsequent decrease in gelation time at higher salt concentrations. The particular effect of CaCl_2_ to speed gelation may be due to its strong destabilizing effect on BSA (Figure 2b), but also could involve ion cross-linking or bridging between proteins providing another aggregation pathway.

We initially hypothesized that slower gelation would lead to material loss and subsequently a weaker, softer adhesive. However, longer crossover times instead produced BSA gels with increased storage modulus (*p* = 0.065, *r* = 0.64, Figure 9e). In the extended gelation process proteins are likely able to aggregate into more ordered structures, forming a more cohesive network. Interestingly, we observe little correlation between the disulfide bond content seen through spectroscopy and the material stiffness. This may imply that changes in gel stiffness may not come from increased covalent bond formation, but structural changes could also be obscured by nonload bearing disulfide bonds or residual cystine within the adhesive. While we did not investigate the curing kinetics of αLa adhesives here, the two most stabilizing salts at 1 M (CaCl_2_ and NaCl_2_) yield stiffer gels than the more neutral salts, possibly indicating a similar trend. Unsurprisingly, the tensile strength of each BSA gels is strongly correlated to its storage modulus (*p* = 0.040, *r* = 0.65, Figure 9f).

We observed a wide spectrum of stabilizing and destabilizing effects when combining the four salts and two protein systems in this study. The concentration where an ion shifts from a stabilizing to destabilizing effect on proteins depends on both the ion and the protein. BSA, as an osmolyte in the bloodstream, is relatively large (66.5 kDa), flexible, and stable across a wide range of ion concentrations.^75,76^ The addition of even large quantities of NaCl stabilizes BSA (increasing *T*_m_), a phenomenon which has been previously attributed to the screening of negative charges within the protein that reduces internal electrostatic repulsion.^77^ All other salts had destabilizing effects on BSA, an effect that we attribute to the chaotropic effects of these cations in solution. Notably, the divalent cation Ca^2+^, which is known to be particularly disruptive, had the greatest destabilizing effect on BSA.^37,78^

αLa, a smaller (14 kDa), more compact protein, is highly dependent on Ca^2+^ ions to maintain its native structure,^57,79–81^ which likely drives to the observed increase in thermal stability with ion concentration. While we expect the vast majority of protein here to already have a bound calcium ion (holo state), the addition of CaCl_2_ likely further shifts the binding equilibrium and thus reinforces the protein’s native structure. It is known that other divalent and even monovalent ions can occupy αLa’s Ca^2+^ binding site and stabilize the protein, which accounts for the similar (albeit weaker) increase in *T*_m_ with NaCl, KCl, and MgCl_2_.^57,82,83^ Many of the ions studied had comparable or stronger effects on the thermal stability of BSA and αLa than the canonical denaturant urea at the same concentration, which reveals their potential to influence the formation of adhesive protein gels.

The development of protein-based underwater adhesives offers significant advantages for both biomedical and structural applications, for example improved biocompatibility or better wetting to and water removal from hydrophilic surfaces. Unlike polymeric materials, where physicochemical relationships allow control over material properties, such relationships are poorly understood for protein-based materials. In this study, we show that the simple addition of salts can greatly affect gelation time, water content, and storage modulus of two scalable protein systems, expanding our toolbox to control these materials. In the process, we also revealed unexpected correlations between protein aggregation and the mechanics of the resulting hydrogel.

While we have focused here on exploring the effects of the four major cations in seawater on the formation of protein adhesives, further investigation of other environmental effects (e.g., sulfate anions or synergistic effects between ions) and the changes to adhesive nano- and microstructure will be important for further developing these materials. While the inclusion of salts in an initiator clearly influences early stage gel formation, the immersion of adhesives into media of varying salinity (e.g., physiological, oceanic, brackish) will change the resulting gel. Understanding and mitigating these changes will be crucial for the reliability of hydrogel adhesives in diverse solution environments.

## 5 Conclusion

In this work we investigated the effect of common seawater salts NaCl, KCl, MgCl_2_, and CaCl_2_ on two barnacle-inspired protein underwater adhesives. Exposure to salt affects the adhesive from the initial denaturation of the protein to the gel formation and the stiffness and bond strength of the cured material. Adhesives made from both BSA, which tended to be destabilized by the salts studied, and αLa, which was stabilized by all of the salts, showed improved properties by exposure to certain salt conditions. The incorporation of salt at lower concentrations (0.1 M) tended to positively impact material properties by slowing gelation, leading to increased stiffness and adhesion, while high concentrations of salt (1 M) substantially weakened the adhesives in most cases. Adhesion tended to improve in comparison to DI H_2_O, but without strong correlation to disulfide bond content of the material. At similar ionic strengths the formation of stronger cohesive networks appeared to come at the expense of interfacial interactions. The links between aqueous chemistries and bulk material properties established in this study will be crucial in developing and optimizing future aqueous underwater-curing protein materials.

## Supplementary Material



## Notes

Representative images of large adhesive samples; oscillatory rheology strain sweep response of BSA gels; lap shear testing molds/curing environment; storage modulus over time for BSA and αLa; ultimate tensile strain of BSA adhesives after curing; Raman spectra for αLa protein gel disks after 8 days of curing; lap shear strength vs storage modulus of BSA and αLa adhesives; lap shear strength vs water content of BSA and αLa adhesives (PDF)

## ⬡

Department of Chemistry, US Naval Academy, 572M Holloway Road, Annapolis, Maryland 21402, United States

## ∥

Psylotech, Evanston, Illinois, 60201, United States

## ∇

Code 6176, US Naval Research Laboratory, 4555 Overlook Ave SW, Washington, District of Columbia 20375, United States

## ○

Code 6920, US Naval Research Laboratory, 4555 Overlook Ave SW, Washington, District of Columbia 20375, United States.

## Abbreviations

(ABS): acrylonitrile butadiene styrene
(αLa): α-lactalbumin
(ASW): artificial seawater
(BAC): benzalkonium chloride
(BSA): bovine serum albumin
(DI): deionized
(l-cys): l-cysteine
(DSC): differential scanning calorimetry
(PC): polycarbonate
(PTFE): polytetrafluoroethylene
(PVC): polyvinyl chloride
