# Supplementary material for: Effect of Salts on the Aggregation and Strength of Protein-Based Underwater Adhesives
Source: ACS Omega. 2025 Nov 5;10(45):54535–48. doi: 10.1021/acsomega.5c07638 (PMC12631481; doi:10.1021/acsomega.5c07638)
Supplement: Supplementary file 1 [file ao5c07638_si_001.pdf]

Supporting Information

Effect of salts on the aggregation and strength of protein-based underwater adhesives

Zachary D. Lamberty[1†], Chloe M. Skogg[1,2†], Michael C. Wilson[1], Maryssa A. Beasley [1], Abdon A. Vivas Tejada[1,2], Beulah A. Peters[1,2], Christopher R. So[1], Elizabeth A. Yates[1,2*]*

1: US Naval Research Laboratory, 4555 Overlook Ave SW, Washington, DC, 20375, USA

2: United States Naval Academy, 572M Holloway Road, Annapolis, Maryland, 21402, United States

[†] Contributed equally

***Corresponding authors:**

Christopher R. So, e-mail: christopher.r.so.civ@us.navy.mil

Elizabeth A. Yates, e-mail: eyates@usna.edu

>3V03_1| Serum albumin | Bos taurus

DTHKSEIAHRFKDLGEEHFKGLVLIAFSQYLQQCPFDEHVKLVNELTEFAKTCVADESHAGCEKS
LHTLFGDELCKVASLRETYGDMADCCEKQEPERNECFLSHKDDSPDLPKLKPDPNTLCDEFKAD
EKKFWGKYLYEIARRHPYFYAPELLYYANKYNGVFQECCQAEDKGACLLPKIETMREKVLTSSA
RQRLRCASIQKFGERALKAWSVARLSQKFPKAEFVEVTKLVTDLTKVHKECCHGDLLECADDR
ADLAKYICDNQDTISSKLKECCDKPLLEKSHCIAEVEKDAIPENLPPLTADFAEDKDVCKNYQEA
KDAFLGSFLYEYSRRHPEYAVSVLLRLAKEYEATLEECCAKDDPHACYSTVFDKLKHLVDEPQN
LIKQNCDQFEKLGEYGFQNALIVRYTRKVPQVSTPTLVEVSRSLGKVGTRCCTKPESERMPCTED
YLSLILNRLCVLHEKTPVSEKVTKCCTESLVNRRPCFSALTPDETYVPKAFDEKLFTFHADICTLP
DTEKQIKKQTALVELLKHKPKATEEQLKTVMENFVAFVDKCCAADDKEACFAVEGPKLVVSTQ
TALA

Table S1. BSA sequence. Sequence of bovine serum albumin, from Protein Data Bank entry 3V03 [rscb.org; Berman, H. M.; Westbrook, J.; Feng, Z.; Gilliland, G.; Bhat, T. N.; Weissig, H.; Shindyalov, I. N.; Bourne, P. E. The Protein Data Bank. *Nucleic Acids Res* **2000**, *28* (1), 235-242. DOI: 10.1093/nar/28.1.235].

>1F6S_1 | ALPHA-LACTALBUMIN | Bos taurus

EQLTKCEVFRELKDLKGYGGVSLPEWVCTTFHTSGYDTQAIVQNNDSTEYGLFQINNKIWCKDD
QNPHSSNICNISCDKFLDDDLTDDIMCVKKILDKVGINYWLAHKALCSEKLDQWLCEKL

Table S2. αLa sequence. Sequence of bovine αLa, from Protein Data Bank entry 1F6S [rscb.org; Berman, H. M.; Westbrook, J.; Feng, Z.; Gilliland, G.; Bhat, T. N.; Weissig, H.; Shindyalov, I. N.; Bourne, P. E. The Protein Data Bank. *Nucleic Acids Res* **2000**, *28* (1), 235-242. DOI: 10.1093/nar/28.1.235].

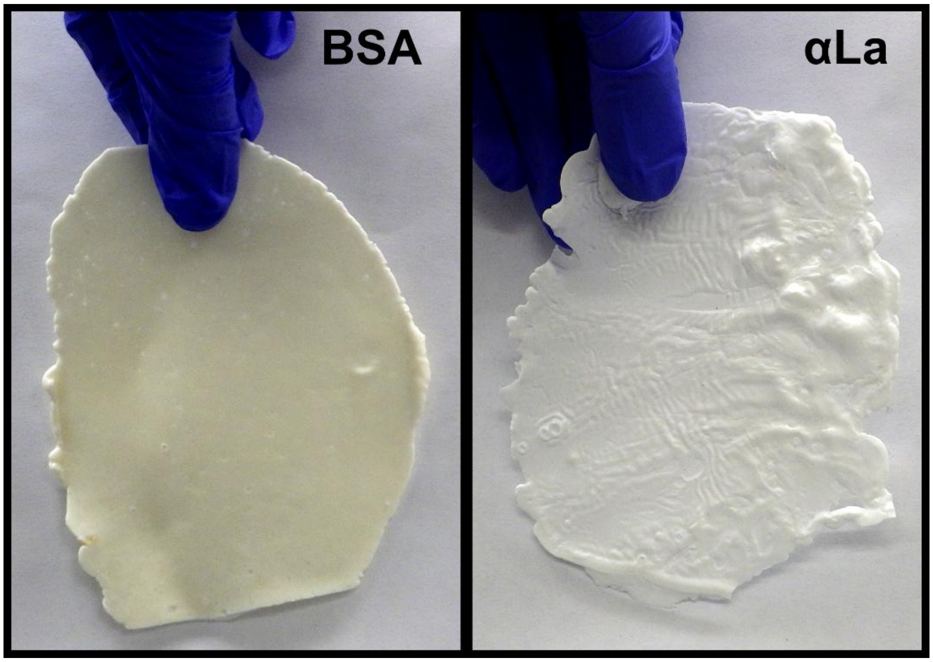

Figure S1. Image of representative large adhesive sample disks for bovine serum albumin (left) and α-Lactalbumin (right). These images represent the overall cured protein gels made after 24 hours that were cut into smaller (15/16") disks for post-cure gel moduli testing, water content, and tensile testing. No holes or defects are present in the cured gels.

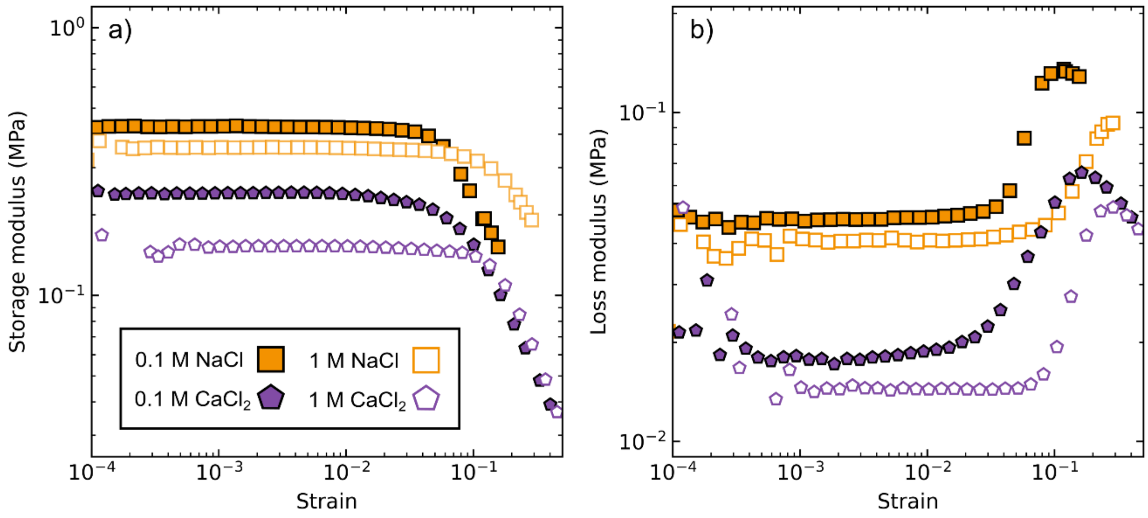

Figure S2. Oscillatory rheology strain sweep response of BSA gels. Representative storage modulus (a) and loss modulus (b) curves of BSA gels in 0.1 M NaCl (filled orange squares), 1 M NaCl (open orange squares), 0.1 M $CaCl_2$ (filled purple pentagons), and 1 M $CaCl_2$ (open purple pentagons). Average values were taken over the linear portion of the curves. Rheology was performed on hydrated gels at room temperature using stainless steel parallel plates. Samples have an applied 50 N compressive load, and strain was varied from 10^{-6} to 10^{-1} at a frequency of 1 Hz.

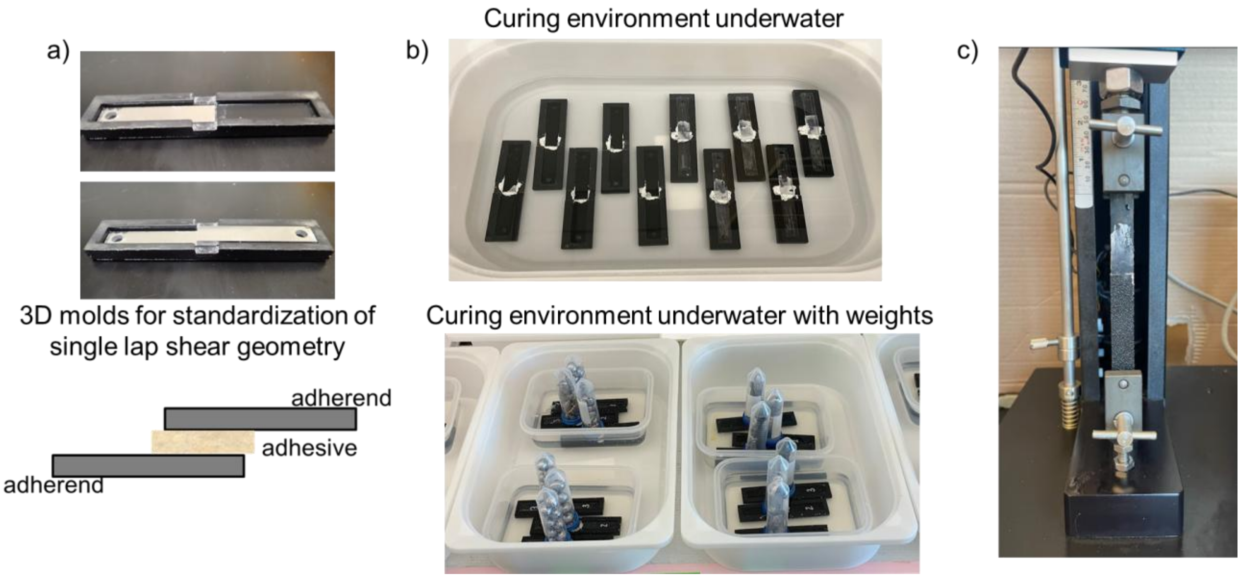

Figure S3. Lap shear sample testing. a) 3D molds printed and used for standardization of single lap shear geometry (0.015 in. thick gap between adherends), b) Curing environment underwater (in specified solutions) with 250 g. weights placed on the overlap in the molds, c) Image of lap shear setup on ADMET eXpert tensile tester at US Naval Academy.

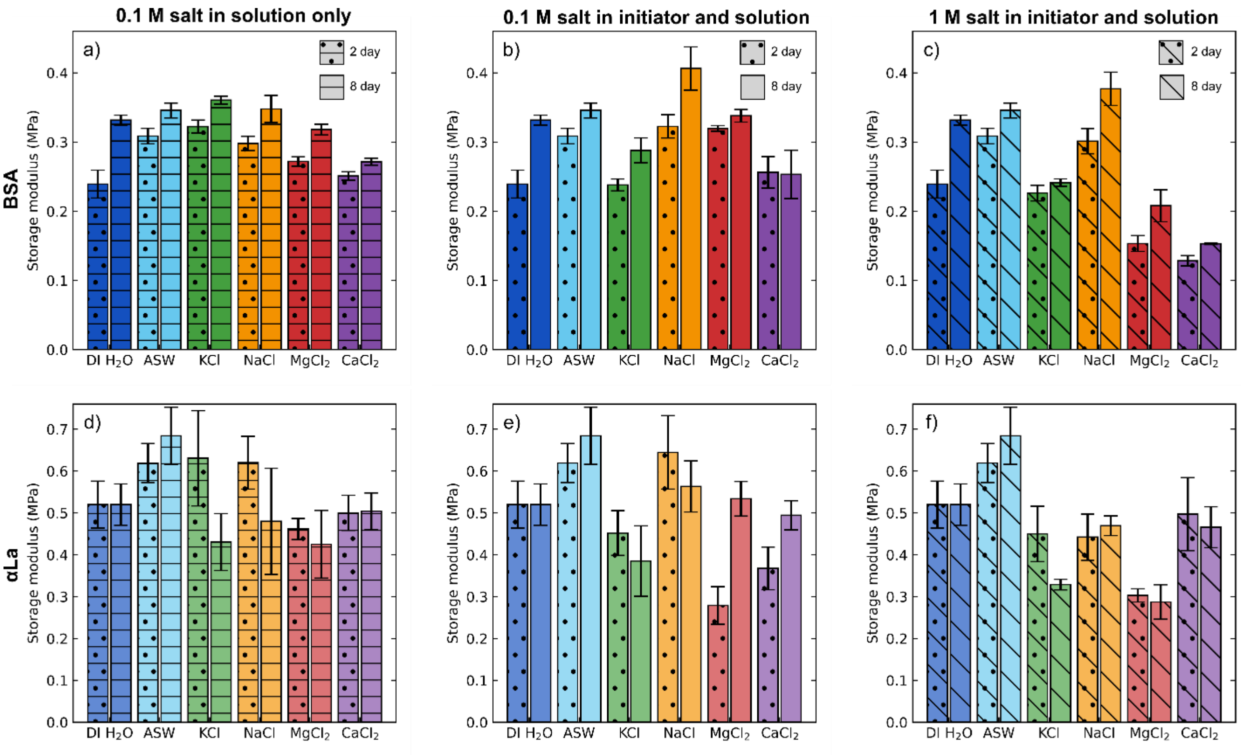

Figure S4. Storage modulus over time. Storage modulus of BSA (a-c) or αLa (d-f) gels after 2 days or 8 days of aging, measured through oscillatory shear rheology of cured adhesive gels. Adhesives are exposed to 0.1 M salt in solution (S condition, a,d), 0.1 M salt in solution and initiator (S/I condition, b,e), or 1 M salt in solution and initiator (S/I condition, c,f). Rheology was performed on hydrated gels at room temperature using stainless steel parallel plates. Samples have an applied 50 N compressive load, and strain was varied from 10^{-6} to 10^{-1} at a frequency of 1 Hz. At least 3 samples were tested per condition; error bars indicate the standard error of the mean.

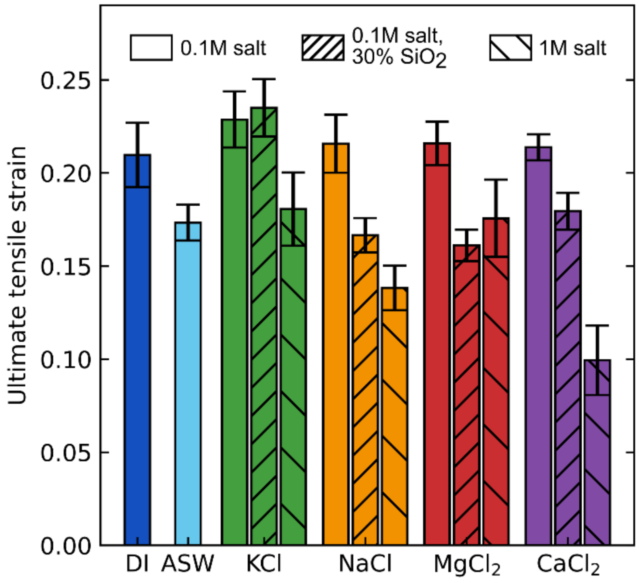

Figure S5. Ultimate tensile strain of BSA adhesives after curing. Strain at peak force during tensile testing of hydrated BSA gels following ASTM D1708 after 8 days of soaking in salt solution. Indicated salts were included in both the initiator as well as the aging solution. Samples were loaded at 5 mm/min until failure. Averages represent 4 – 20 specimens and error bars indicate the standard error of the mean. The addition of silica into the gel decreases the tensile strain at failure.

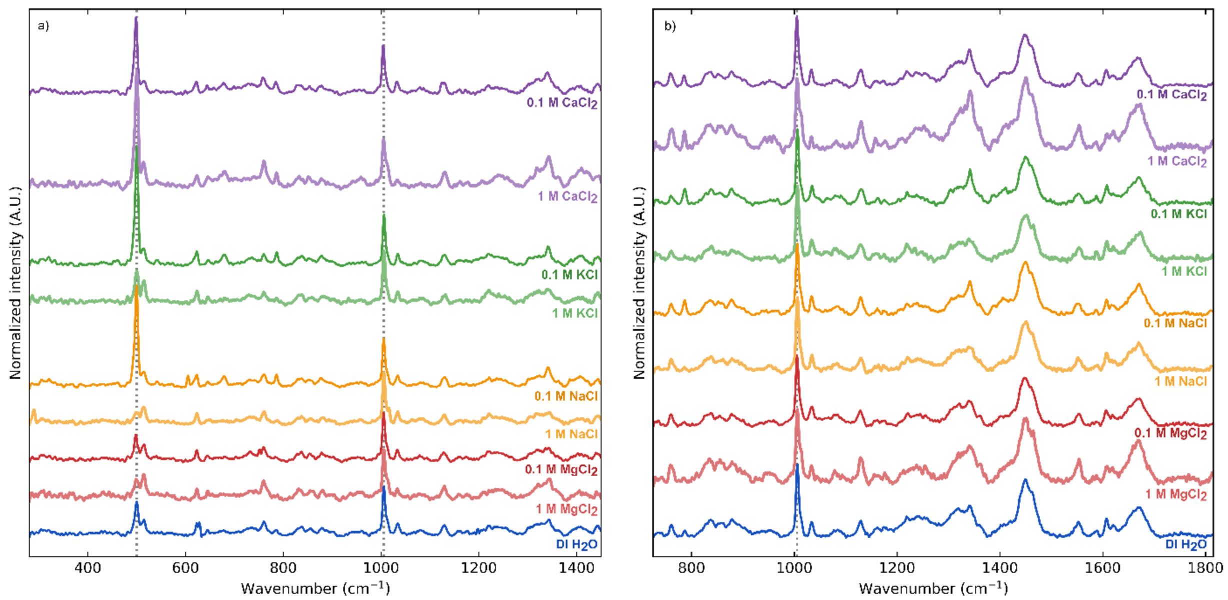

Figure S6. Raman spectra for cured hydrated αLa protein gel disks after 8 days curing. Raman spectra of αLa adhesives exposed to (from bottom to top): DI H_2O, 1 M $MgCl_2$, 0.1 M $MgCl_2$, 1 M NaCl, 0.1 M NaCl, 1 M KCl, 0.1 M KCl, 1 M $CaCl_2$, and 0.1 M $CaCl_2$. Raman spectra were acquired from 280 to 1452 cm^{-1} (a) and 726 to 1813 cm^{-1} (b) (due to range limitations) with 1.5 second integration time and 128 accumulated scans using a Renishaw inVia Raman microscope with a 725 nm diode laser at 188 mW power, a 1200 line/mm grating, and a 50x long working distance objective. Spectra were normalized to the Raman peak at 1005 cm^{-1}.

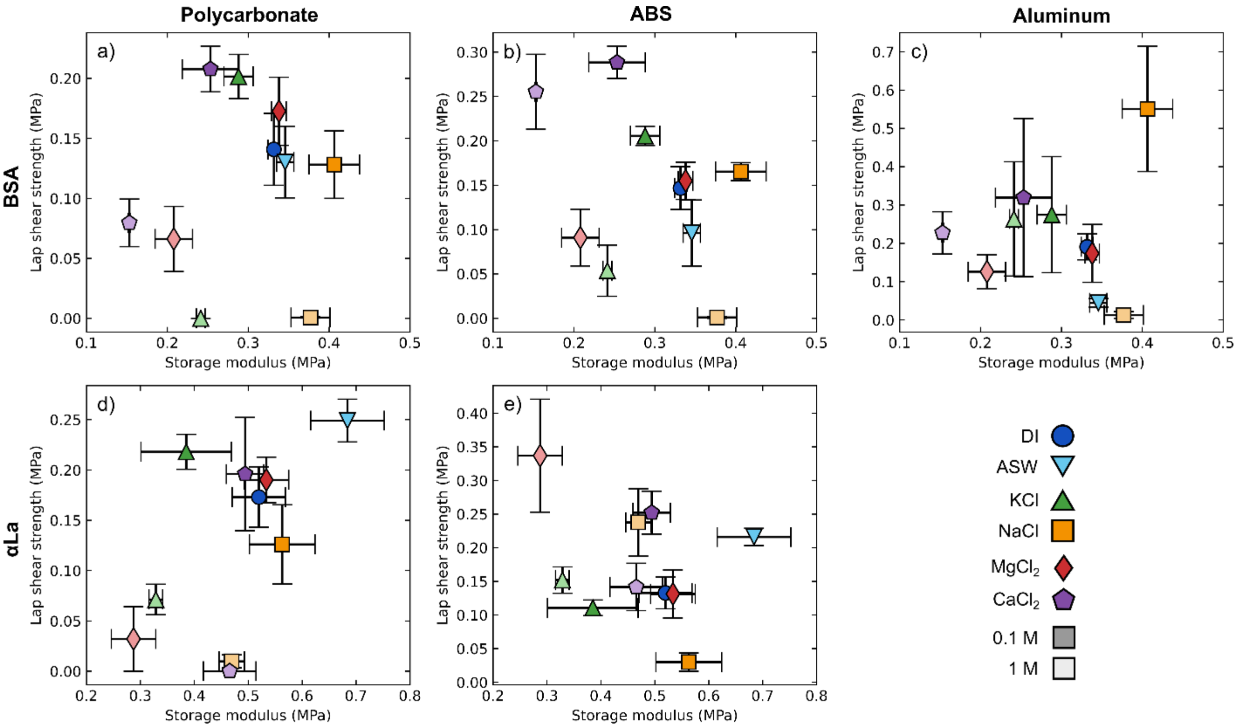

Figure S7. Lap shear vs storage modulus. Lap shear strength vs storage modulus of BSA (a-c) and αLa (d-e) adhesives exposed to the indicated salts on polycarbonate (a, d), ABS (b, e), or aluminum (c). Storage modulus was obtained through oscillatory shear rheology of hydrated gels after 8 days of curing, using stainless steel plates in a parallel geometry. Samples have an applied 50 N compressive load, and strain was varied from 10^{-6} to 10^{-1} at a frequency of 1 Hz. At least 3 samples were tested per condition; error bars indicate the standard error of the mean. Lap shear strength was obtained following ASTM D1002 on samples with a 0.5" x 0.5" overlap and 0.015" adhesive thickness. Samples were aged for 8 days then pulled apart at a rate of 1.5 mm/min while hydrated until failure. Samples were tested in sets of 3 – 6, with error bars representing the standard error of the mean.

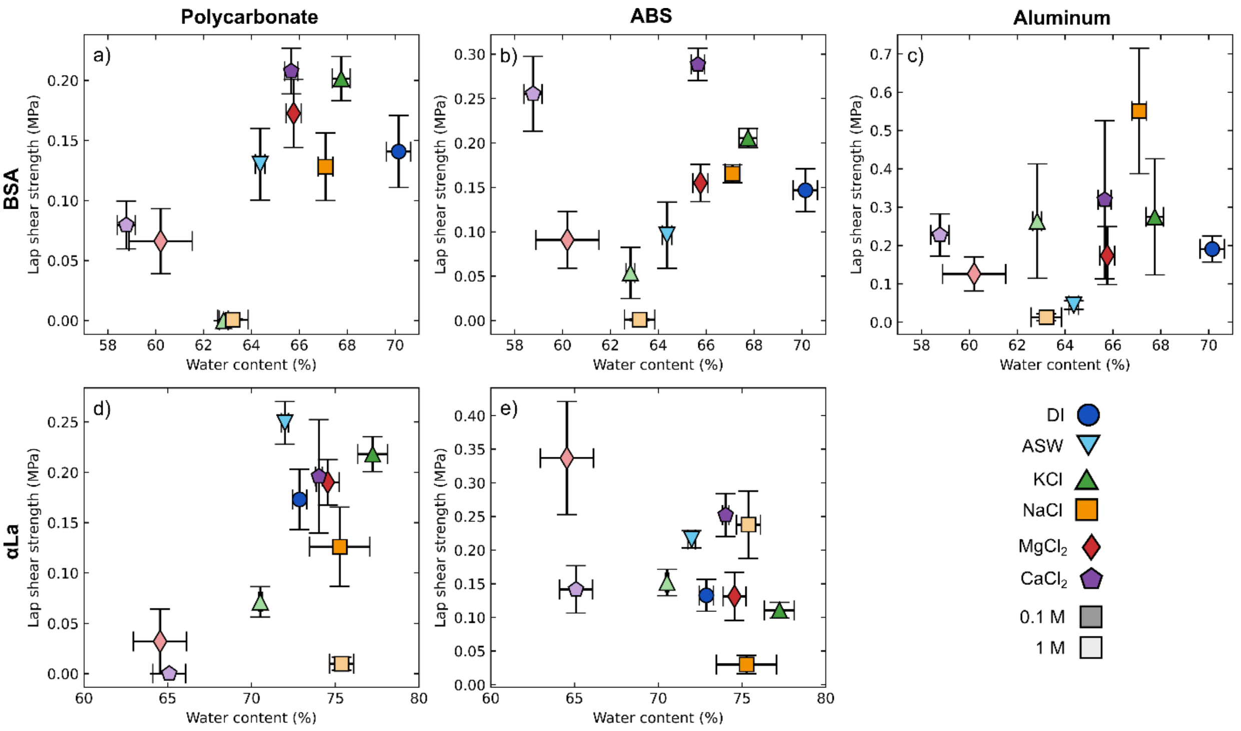

Figure S8. Lap shear vs water content. Lap shear strength vs water content of BSA (a-c) and αLa (d-e) adhesives exposed to the indicated salts on polycarbonate (a, d), ABS (b, e), or aluminum (c). Lap shear strength was obtained following ASTM D1002 on samples with a 0.5” x 0.5” overlap and 0.015” adhesive thickness. Samples were aged for 8 days then pulled apart at a rate of 1.5 mm/min while hydrated until failure. Samples were tested in sets of 3 – 6, with error bars representing the standard error of the mean. Water content of the gels was obtained by first allowing gels to cure in the indicated solution for 8 days, then drying for 1 week in a desiccator followed by 1 week in an oven at 60 °C. The mass loss upon drying was assumed to be entirely water, and water content was calculated by the change in mass during the drying process. Points are the average of between 3 and 13 measurements on individual gels, and error bars indicate the standard error of the mean.
